# Exploring the Hypothetical Impact of Genetic Engineering on Ethnicity: An Analysis of a Large‐Scale Data Set Retrieved From a Museal Setting

**DOI:** 10.1111/bioe.70005

**Published:** 2025-06-25

**Authors:** Niklas A. Döbler, Alexander Pastukhov, Claus‐Christian Carbon

**Affiliations:** ^1^ Department for General Psychology and Methodology University of Bamberg Bamberg Germany; ^2^ Research group EPÆG (Ergonomics, Psychological Æsthetics, Gestalt) Bamberg Germany; ^3^ Bamberg Graduate School of Affective and Cognitive Sciences (BaGrACS) Bamberg Germany

**Keywords:** CRISPR/CaS9, gender, genetic engineering, human enhancement, racism, skin color, stereotypes

## Abstract

Critics of human genetic engineering warn that if ever put into practice, this will diminish human diversity, especially regarding skin color. Nonetheless, given the solid and shameful causal link between skin color and discrimination, the provocative question is whether to manipulate this feature and create children whose stereotype‐aligning features reduce the risk of evoking hostility in the social environment. To address this possibility, we analyzed data from an interactive exhibit in a German museum that partly addresses these questions. Visitors could manipulate randomized features of a virtual child—for example, appearance and intelligence—to align them with their notion of a “perfect child.” Analysis of *N* = 13,641 virtual children showed an apparent effect on aligning skin color with a Caucasian type. This was true for extreme light and dark, randomly assigned initial skin colors, but stronger for the latter. This preference could reflect the attempt to align the hypothetical child's skin color with the creating visitors. We also analyzed the chosen skin‐color‐dependent distribution of designed intelligence based on previous findings showing that high intelligence is less desirable for Black than White persons. We revealed that virtual children with a chosen darker skin color were designed with relatively lower intelligence and a larger proportion of maximized and minimized values. Although most effects were small, they might indicate racial prejudices and/or the attempt to design virtual children with high alignment with normative stereotypes. Our findings provide an important starting point to empirically inform the critical and timely debate about human genetic engineering.

## 1 Introduction

In many countries, individuals with a darker skin tone are likely to experience and suffer from racism and racial prejudice [1, 2, 3, 4]. In this context, skin color is linked to normative stereotypes, that is, convictions about how people should (*prescription*) and should not act (*proscription*) [5]. Not adhering to racial stereotypes can yield a wide range of disadvantages for the norm‐defying individual [6]. Stereotypes have been shown to entail a pronounced action‐guiding component [7, 8, 9]. This yields the effect that external evaluators and the stereotyped individual may choose to affirm stereotype‐matching and punish divergent behavior to minimize expected disadvantages, even if this maintains stereotype content [6].

One evident solution to the injustice of racial stereotypes would be a large‐scale societal intervention that emphasizes the existence and harm these stereotypes do. The success of these more policy‐focused interventions can be seen in the increasing number of elected Black representatives [10] and improved participation in the labor market [11] in the United States of America following the 1965 Voting Rights Act. Although racial injustice is far from being eliminated, one can think of a wide range of possibilities to *adapt the social environment* so that individuals subjected to prejudice and stereotypes are less likely to experience their harmful consequences.

However, instead of changing the environment, one could directly adapt the humans living in this environment [12]. This approach will target the physical features that determine stereotype content. An example of this practice would be the historical and contemporary use of dermatological skin‐lightening products, whose marketing often highlighted the social benefits of a more pale skin tone [13]. In this context, novel and emerging biotechnologies promise a scientifically aided and significantly tighter grip on the biological factors that characterize the valued aspects of the human condition [14]. Emerging genetic engineering technologies like CRISPR/Cas9 [15] are controversial for potentially targeting features that constitute the diversity of humanity [16]. For instance, these interventions enable the modulation of the melanin production of cells [17] and are capable of changing coat color in animals [18]. Hence, there might be a pathway to use genetic engineering to influence human pigmentation. Using technology to adapt the humans themselves and alter the capabilities necessary for coping with the social environment's imposed demands is a practice we understand as *Human Enhancement* [12]. Most importantly, this practice can be carried out in good spirit, yet it must not necessarily live up to its name and fail in its intention to facilitate adaptive and positive results [12].

Imagine human genetic engineering being a probable way to create the infamous “designer babies.” Here, parents are likely to know that certain phenotypic variations and their genetic influence are more likely to be associated with social and physical harm. Hopefully invested in the child's well‐being but contemplating the use of highly transformative technology, do they not have the “moral obligation” for “Procreative Beneficence” [19], that is, “select the child, of the possible children they could have, who is expected to have the best life, or at least as good a life as the others, based on the relevant, available information” [19, p. 415]. Does this entail manipulating the genotype of children to manifest a more stereotypical yet potentially less negative‐consequences‐evoking phenotype?^1^

Implications and extent of this principle have sparked a controversial bioethical debate [20, 21], with Sparrow directly mentioning the case of skin color:

Savulescu, therefore, cannot avoid the conclusion that if technologies of genetic selection for racial markers ever become available, parents in racist societies would be obligated to select against children who might be identified as members of the oppressed race. As a consequence, if parents do as he suggests, the wildest dreams of race supremacists would be realized and racial minorities would disappear in one or two generations.

[21, pp. 51–52]

As shown in the above quote, the unavailability of respective technologies renders this argument preventive. Apart from these theoretical objections, empirical research on the various kinds of Human Enhancement can aim to produce preemptive insights to guide regulatory decision‐making processes [22]. Thus, the present paper investigates how skin color and action‐guiding associated normative stereotypes may influence how people conduct human genetic engineering. Drawing upon data gathered from an interactive, ecological German museum context, we seek to address how genetically adapting ourselves for enhancement purposes may influence human diversity.

## 2 Hypotheses

The action‐guiding power of stereotypes can either be embraced or rejected. Hence, two hypotheses are conceivable. First, the *Good‐Child‐Hypothesis (Good‐CH)*. Following this acknowledges that normative stereotypes, in general, are instructive for being “a good group member” [23, p. 287]. Since not adhering to these normative convictions increases the chance of experiencing adverse consequences [6], people may use genetic engineering to create “good” children, aka, children who embody stereotypes on a genotypical level. Important note: “Good” hereby strictly refers to the evaluative criteria of a society that punishes stereotype‐diverging stereotypes and thus maintains racist prejudice.

Another possibility is that people purposefully choose to design children who counteract stereotypes and create so‐called “vanguards” [6]. The precursive adaptation of their children then serves the goal of modulating the distribution of features that people use to inform their stereotypes. This may be hard since humans are not very skilled with the mathematical integration of (prior) probabilities [24]. Nonetheless, one study showed that if people are told to imagine that males and females occupy social roles with equal frequency, stereotype content about females approximates those about males [25]. Moreover, when confronted with stereotype‐disconfirming individuals, people tend to generalize observed features to the respective group [26]. The goal here is to create children whose adapted features may induce an adaptation of the environment according to non‐racist values. This reflects the optimistic position that human genetic engineering can help to emancipate disadvantaged children and lead to generally beneficial outcomes for humanity [27]. We call this the *Emancipate‐Child‐Hypothesis (Eman‐CH)*. Although the hypotheses were mainly drawn from the literature on gender stereotypes [28], we argue that their basic logic applies to any social context in which adherence to stereotypes is key for preventing maladaptive states. This is generally important in the case of embryonic genetic engineering or any similar intervention with unborn or very young humans; this particular being is not an agent that could decide whether to follow the stereotype content [6]. Instead, this content is inscribed into the genetic reality by active parents/designers. So, the left marks of the stereotype content may be identifiable even if they never manifest in phenotypical variation [28].

## 3 The Present Study

We have obtained a large data set containing *N* = 13,643 virtual children, all created at an interactive exhibit about creating one's “perfect child.” The exhibit's location is the “*Deutsches Museum Nürnberg—Das Zukunftsmuseum*” [Museum of the Future Nuremberg]. Visitors could approach a large touchscreen with the following instructions: “Create your perfect child! You have €250,000 at your disposal. Weigh up which aspects are particularly important to you.” Visitors could now spend the virtual money. After potentially selecting the child's gender, they could change the skin, hair, and eye color of the virtual offspring. They could then decide to add or eliminate genetic disease dispositions (e.g., for cancer or diabetes) and manipulate five personality traits and four “talents” (musicality, intelligence, creativity, sportiness).

Each child was assigned random values in all manipulable features. Distributions generated from these values did not stretch beyond the whole feature range. Importantly, visitors paid for *change* but not the *range of change*, so they had no economic benefit from not maximizing a trait or decreasing/minimizing another.

After completing the exhibit (2–10 min), people were informed that environmental influences affected the manifestation of their manipulations. Most frequent choices of other visitors for manipulated traits, color choices, and most valued feature expressions were communicated to them, but without any statement of the extent to which a specific value was associated with skin color, etc. Hence, visitors only got a broad overview and no information about what the prototypical “White” child, for instance, was like. The didactic concept of the exhibit is to convey the power and limitations of human genetic engineering.

Visitors were not informed that their behavior would be potentially subjected to scientific inquiry. However, the exhibit stored no personal information, making the data set fully anonymous. Written consent to use the data was obtained by the museum. This study was reviewed and approved by the Ethics Committee of the University of Bamberg, Germany (Dossier number 2023‐07/31).

The present inquiry will focus on one controversial racial stereotype: intelligence. Human history is characterized by a strong racialization of intelligence, with the dominant “Western” modes of expression being the viewing of darker skin types as less intelligent than lighter ones [29]. Linked to this idea are highly received books that argue for a strong genetic basis of identified differences in intelligence across ethnic groups and people of color, for example, “The Bell Curve” [30] or “Germany Abolishes Itself: How We're Putting Our Country in Jeopardy” [31]. Needless to say, these books were highly controversial.^2^

We are aware that this topic is extremely controversial. Our hypotheses are confined to how people *perceive and value* intelligence, conditional on ethnicity and associated phenotypes. Our results and this investigation should neither be read as confirming assumed intelligence differences across ethnic groups nor as a statement that among the genes that determine skin color, some are inherently “better” than others. This investigation concerns the intricate action‐guiding effect of stereotypes. Some may be inherently harmful, yet people may believe that not adhering to them may be worse than the initial harm.

## 4 Predictions

Given evident racial discrimination based on darker skin color in Germany, the country where this study was conducted, we predict that a darker initial, randomly assigned skin color will yield a higher probability of motivated change (Prediction 1), with the outcome of all changes predicted to be a stereotypical German, that is, Caucasian skin type (Prediction 2). In short, following the logic of creating a stereotypical, aka “good” German, people whose virtual children are randomly assigned a darker skin type are expected to change and align it with the prevailing stereotypical White appearance. The Eman‐CH does not predict any pronounced preferences.

A recent study has revealed the presence of normative racial stereotypes for intelligence [5]. Supporting Information (p. 180) of Hudson and Ghani's study showed that participants rated intelligence as highly prescriptive for all races (Black, Latino, Asian, Middle Eastern, White). However, in direct comparison, being intelligent was *more* desirable for White and Asian persons than it was for Black persons. Moreover, intelligence was preferable for White than for Middle Eastern or Latino persons. There were no statistically significant differences in prescription strength between Black persons and Middle Eastern or Latino persons [5]. Hence, under both hypotheses, people should aim for high intelligence values (Prediction 3). Yet, if people decide to create a child with a darker skin tone, the Good‐CH predicts a negative relationship between skin darkness and intelligence. Given the general prescription of high intelligence, the Eman‐CH predicts that intelligence is equally high for all groups (Prediction 4). See Table 1 for an overview.

**Table 1:** Hypothesis and predictions regarding the examined data.

		Prediction for the examined data			
		Probability of changing skin color	Designed skin color (if changed)	Designed level of intelligence	Association between designed intelligence and darker skin color
Hypothesis	Good‐GH	More likely for darker skin	Orientation to a Caucasian norm	High	Negative
	People are guided by normative stereotypes, e.g., to minimize adverse consequences for their children.				
	Eman‐GH	No difference	No pronounced preference	High	None
	People create stereotypes disconfirming children, e.g., to create a more egalitarian future.				

## 5 Methods

In the raw data set, skin color and intelligence choice were present as values between −1 and +1, based on the chosen position of a virtual slider on the touchscreen. Concerning the color choice, the slider position was then related to RGB color values in the data.

Inspection of these variables led to the exclusion of the first two data points of the data set. This was because of apparent irregularities in the relationship between RGB‐coded skin color and virtual slider value. In these two cases, the same slider value produced largely different skin colors than in the remaining data set. We then reconstructed color hexadecimal color codes (hex codes) from RGB values as present in the data. This method was slightly imperfect, probably due to the inconsistent amount of coded decimal numbers and rounding procedures. The same color slider value could yield diverging RGB values and, thus, different hex codes. However, visual inspection of the colors yielded no relevant differences. Overall, there might be a slight yet insignificant variation in the data's hex code‐slider value relationship. After the described exclusions, the analyzed data set contained *N* = 13,641 virtual children. We have no information on how many visitors were responsible for this amount, nor any socio‐demographic information about them.

Virtual children could be “born” with one of four predefined skin colors. Their coding as Type 1–4, and case numbers are visible in Table 1. This initial skin color was assigned randomly *χ* ^2^(3) = 1.63, *p* = 0.65, and could be changed by the visitors for 10,000 virtual euros to any value present at the slider.

Assigned intelligence was uniformly distributed between −0.40 and 0.40, but could be manipulated down/up to −1 and +1 for 10,000€. Analysis of variance (ANOVA) revealed a non‐equal distribution of randomly assigned initial intelligence across assigned skin color, *F*_(3, 13637)_ = 2.62, *p* = .049. Post‐hoc *t‐*test with Holm adjusted *p*‐values found a significant difference in assigned intelligence and assigned skin color among the two darkest skin tones (Mean_Type 4_ = 0.010 > Mean_Type 3_ = −0.005). Differences with other Types (Mean_Type 1_ = 0.000, Mean_Type 2_ = 0.001) were nonsignificant (*p*'s > 0.05). We found no significant correlation between the chosen skin color slider value and assigned intelligence values, *r* = 0.01, *t*_(13639)_ = 1.02, *p* = 0.302. The ANOVA with only those virtual children included that retained assigned skin color showed no difference in assigned intelligence *F*_(3, 2683)_ = 0.69, *p* = 0.56. Hence, we assume the found difference to be a statistical artifact.

Bernoulli logistic regression (Coefficient‐prior: *N*(0, 1)) was calculated to evaluate the probability of changing randomly assigned initial skin color (Table 1). All other settings retained the default of the used *brms* [32] package. Since appearance manipulation was the second screen and people had little opportunity to spend their money except on the first screen's gender selection, available money was not included as a predictor.

To model chosen skin color and intelligence, we chose a zero‐one‐inflated beta regression (ZOIB). This model combines a beta regression with two logistic regressions to model the whole outcome distribution instead of being confined to the mean [33, 34]. Beta regression is meant to model the overall shape of the distribution, while logistic regressions address the probability of a value being 0 or 1. To suit our data for this procedure, we scaled it to 0‐1. After calculating the models, predicted values were reverted to −1;1 to enhance comprehensibility.

**Figure 1:**
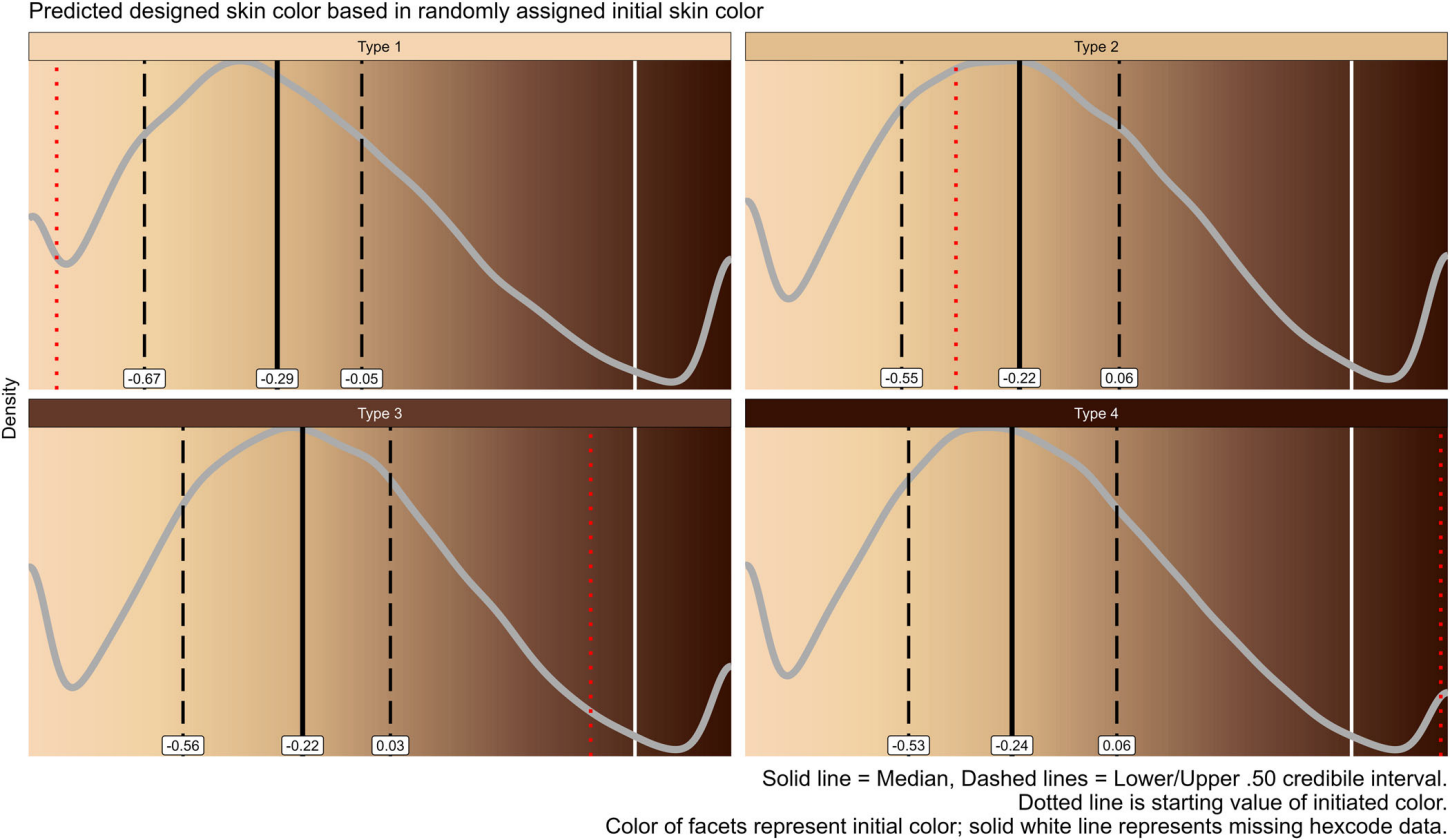
Results from a zero‐one‐inflated beta regression predicting chosen skin color value for each randomly assigned initial skin color Type. Only values from virtual children whose skin color was modified were used (*n* = 10,954). Background reflects skin color as indicated in the exhibit. Gray curve shows the estimated distribution of chosen skin color. Red dotted line shows the initial skin color. Black solid line and black dashed lines show median and 50% credible interval. The white solid line represents missing hexcode data. Density data stems from 20,000 non‐averaged posterior draws for each Type. Regression coefficients can be found in the supplementary. As visible, designed skin color distributions showcase three “bumps,” highlighting extreme values and the modus, which approximates the median.

The designed skin color value was predicted by a categorical randomly assigned/initiated skin color (Prior: *N*(0, 1—Figure 1). All other settings retained the default of the used *brms* [32] package. The model with the chosen level of intelligence as the outcome used the chosen skin color slider values as a predictor for all model coefficients (Prior: *N*(0, 1)). All other settings retained the default settings of *brms*. ZOIB models only used data from virtual children whose outcome of interest was actively changed, aka, designed but maintained data from virtual children whose predictor (skin color) was not changed. This was done to eliminate purely random and computer‐generated variation in the outcome. Hence, respective outcomes are referred to as *designed*. At the same time, data on features containing virtual children whose randomly assigned values were chosen to be maintained (e.g., did not change skin color) will be called *chosen* (usually the whole data set).

If not stated otherwise, results discuss posterior predictive distributions, i.e., averaged draws from the modeled posterior to communicate the expected value of the modeled distribution [34, 35, 36].

## 6 Results

When interpreting the following results, consider that we had to reconstruct the hex color codes from the data and that lightning in the museum and hardware specifications of the used screen may have influenced the color perception. Nevertheless, a general distinction of skin darkness as a continuum is possible.

### 6.1 Prediction 1

Overall, randomly assigned initial skin color of 80.30% (*n* = 10,954) of the virtual children was changed. Table 2 shows results from the logistic regression, while Table 3 features comparisons of the change probability. Both indicate a higher change probability for virtual children whose randomly assigned initial skin color was darker than for those with lighter skin.

**Table 2:** Overview of randomly assigned and initiated skin colors and prediction of changing/designing it.

Randomly assigned initial skin color	*n*	Probability that skin color was changed	
(hex color codes)		Expected value (Median)	CI (95%)
Type 1 #F2D3AF	3427	77.80%	76.5%. 79.3%
Type 2 #DEBB8D	3424	70.30%	68.9%. 71.8%
Type 3 #613928	3443	87.20%	86.2%. 88.4%
Type 4 #361103	3347	86.00%	84.8%. 87.1%
Observations	13641		

*Note:* Color of the column and hex code represent the randomly assigned initial skin color. The probability that skin color was changed (expected value and CIs) was calculated using posterior predictive values. Regression coefficients can be found in the supplement.

Abbreviation: CI, credible interval.

**Table 3:** Marginal effects of change/design probability based on randomly assigned initial skin color.

Randomly assigned initial skin color		Group 1–Group 2		
Group 1	Group 2	Median (pp.)	CI (95%)	Maximal probability of effect
Type 1	Type 2	0.08	0.06. 0.09	100%
Type 1	Type 3	−0.09	−0.11. −0.08	100%
Type 1	Type 4	−0.08	−0.10. −0.06	100%
Type 2	Type 3	−0.17	−0.19. −0.15	100%
Type 2	Type 4	−0.16	−0.18. −0.14	100%
Type 3	Type 4	0.01	0.00. 0.03	93.40%

*Note:* Groups 1 and 2 refer to contrast, so that negative values for the difference indicate a higher probability of color change for group 2. Colors of cells represent randomly assigned initial skin color. MPE is a Maximal Probability of Effect: A probability that a parameter is strictly positive or negative. It is a measure of statistical significance of the effect, with higher values indicating stronger evidence for statistically significant results.

Abbreviations: CI 95, 95% credible interval; pp., percent points.

### 6.2 Prediction 2

Figure 1 shows the predicted designed color values for each initial skin color from Type 1 (least dark) to Type 4 (darkest). Regression coefficients (Supplement) reveal some variations, for example, in proportion of maximized and minimized values. Nonetheless, distributions mostly aligned with the median, settling at a more Caucasian skin tone.

### 6.3 Predictions 3 and 4

**Figure 2:**
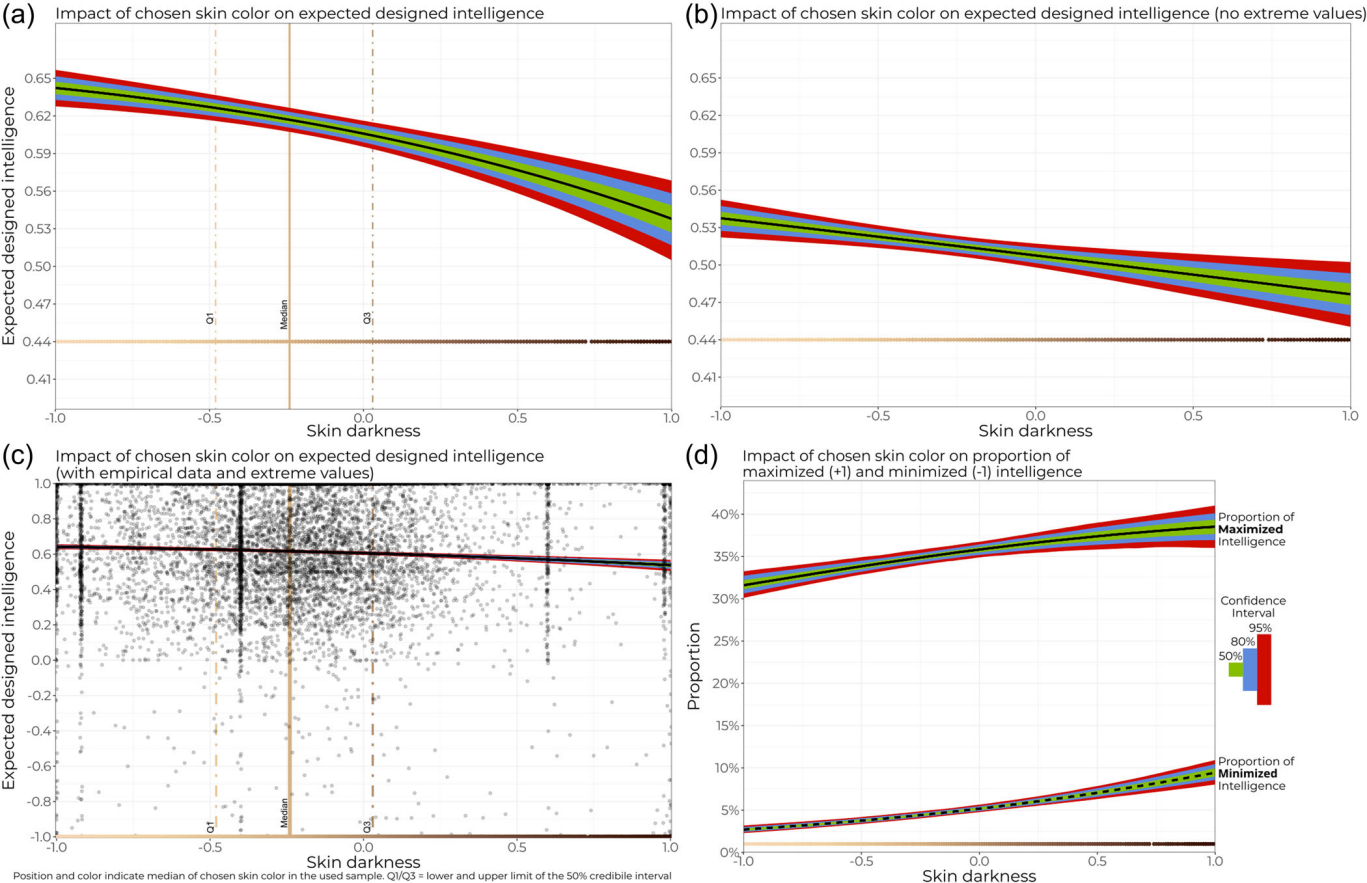
(a) Designed intelligence as a function of chosen skin color (excluding participants who did not modify intelligence), a zero‐one‐inflated beta regression (*n* = 10,738). (b) Designed intelligence as a function chosen skin color (excluding participants who did not modify intelligence) and without extreme (0/1–−1;1) values, beta regression (*n* = 6488). (c) Predictions of zero‐one‐inflated beta regression (a) overlayed on empirical data (jittered for visibility). Vertical stripes refer to people who choose to maintain randomly assigned initial skin color. (d) Proportion of extreme values calculated from the zero‐one‐inflated beta regression model shown in (a). Note that in this case, a higher proportion of maximized values increases and a higher proportion of minimized values decreases the mean value of the distribution. Colored line at the bottom represents skin tone as presented at the exhibit, with the gap representing missing hex color code data. Information on parameter estimates can be found in the supplement.

Of all virtual children, 78.71% (*n* = 10,737) received a modification in randomly assigned intelligence. We evaluated expected posterior values and found a negative relationship between chosen skin darkness and designed intelligence (Figure 2a). However, the effects were relatively small given the maximum possible difference of ±2. Overall, people designed their virtual children with high levels of intelligence and preferred maximizing this value over minimizing it (Figure 2c,d). To test for possibly exaggerated stereotypes, we also explored the impact of chosen skin color on the proportion of designed maximized/minimized values. We found a positive relationship with both proportions (Figure 2d). To test whether extreme values caused the link between skin tone and designed intelligence, we conducted an ordinary beta regression with the same configuration as the zero‐one inflated one, but eliminated all minimized/maximized (−1, 1) data points. Results confirmed the initial analysis (Figure 2b).

## 7 Discussion

Human genetic engineering promises widespread, radical, and controversial transformation of the human condition [37]. At the same time, humans are constantly subjected to social evaluation, the results of which can yield adverse outcomes. In coping with encountered normative stereotypes, we suggested that people may exploit the transformative capabilities of genetic engineering to ‘adapt their children instead of the environment’ [12] and design virtual children in a way that minimizes the probability of racial discrimination and potential negative experiences caused by them violating stereotypes, that is, the Good‐GH [6]. Given that people are keen to point out the potential benefits of genetic engineering [27], we also considered the possibility that people purposefully design virtual children whose genetic makeup contradicts stereotypes (Eman‐CH). One assumed goal is to create enough social friction that socially shared stereotypes are revisited and adapted [6, 25, 28].

Putting these hypotheses to an empirical test, we analyzed a large data set from an interactive “design‐your‐perfect‐baby” exhibit in a German museum. We focused on the potential discrimination due to darker skin color in general and the findings [5] that high intelligence is less predictive for Black persons than for white persons. Our results indicate that if people are confronted with a prefigured virtual baby and are asked to manipulate it so that it resembles their idea of a “perfect” child, feared racial discrimination and normative, skin color‐dependent stereotypes may guide their decision‐making.

We found that if assigned an initial baby with darker skin, people were more likely to change their virtual babies' skin color. Regardless of the randomly assigned initial skin color, people designed their virtual babies to have a skin color close to what may be considered a Caucasian norm. This may be interpreted in a way that people design their “perfect” hypothetical children in a preventive manner, that is, choose skin color so that racial discrimination in Germany is less likely. This would be what is expected under the Good‐CH. However, even under the Eman‐CH, people can prefer one skin tone more. The critical factor for the latter is whether associated normative stereotypes are enacted or dismantled.

People generally valued higher intelligence for their virtual children. The exhibit was programmed so that people always paid the same amount of virtual money to change their child's features, regardless of the range of change. Hence, people had no economic reason to reduce or constrain intelligence values to lower levels and not maximize them. However, people seemed to value this trait slightly less for virtual children chosen to have darker skin. This is, visitors *did not* design these virtual children as “dumb,” but simply less intelligent than virtual children with lighter skin and not as universally intelligent as possible. Supporting recent findings on the ethnicity‐conditional desirability of intelligence [5], evaluating designed intelligence at the line of chosen skin color provides preliminary evidence for the Good‐CH.

However, there are alternative interpretations to our findings. First, skin color choice could be driven not by the goal of preventing hypothetical racial discrimination but by aligning the child's skin color with the skin color of the designing visitor. About one‐quarter of the population in Germany comes from a family with a migration history [38]. We can only speculate about the ethnic distribution of the visitors. However, it should not be too farfetched to state that Black persons or People of Color are minority groups in Germany. This may explain decision‐making based on randomly assigned initial skin color and subsequent maximization efforts, but not the general negative association between skin darkness and chosen intelligence.

Apart from the enactment of normative stereotypes, the anonymous setting of the exhibit, in conjunction with its gamified programming, may have inclined people to exaggerate racial stereotypes up to the point where some of them designed a blatantly racist stereotype. Motivating to express these stereotypes runs counter to the educational purpose of this station.^3^ Nonetheless, this disturbing possibility is supported by findings on the positive relationship between minimized designed intelligence values and skin darkness, but contradicted by the positive relationship between maximized designed intelligence and skin darkness. Eliminating extreme values from the data set did not eliminate the negative impact of chosen skin color on designed intelligence. Here, statistical analyses should investigate the potential presence of distinct groups within the chosen skin color and their potential diverging alignment with the hypotheses. In general, many explanations are possible for the patterns we found in our data. A definitive conclusion about the precise motivation of visitors, including their held stereotypes, demands a more thorough investigation in a more controlled setting.

It may be questioned why people at the exhibit were able to manipulate skin color in the first place. Does this not inevitably lead to the virtual “physiological” manifestation of socially shared stereotypes?^4^ Our rebuttal is that *not* inviting visitors to contemplate the skin color preferences of their “perfect” child would be negligent of one of the most controversial topics in the debate about interventions that target the human embryo [see 21]. Doing so would run counter to the educational purpose of the exhibit. Regulatory decisions in this regard must be informed by science and public opinion. This requires research that assesses the possibly controversial employment of the former's products, fueled by controversial examples of the latter. The presence of harmful stereotypes is nothing to be proud of, but mitigating their negative consequences requires a large‐scale analysis of their manifestations across present and future contexts.

## 8 Limitations

Most evidently, the ecological setting limits the interpretability of our findings. We have no information about visitor demographics, psychometrics, or their seriousness in engaging with the exhibit. Moreover, we do not know how many visitors have engaged with the exhibit in total. In addition, the exhibit was not constructed to serve as psychological experiment to serve an educational purpose.

Hudson and Ghani [5] evaluated racial stereotypes using neutral descriptions like “Black/White person.” Nonetheless, they found a substantial intersectionality in terms of stereotypes based on skin color, gender, and sexual orientation. Gender was also manipulable at the station, but was not the focus of this analysis, and neither were other appearance features like hair and eye color. To investigate the whole extent of the potential action‐guiding effect of stereotypes, future research should investigate potential interaction effects while extending toward features other than intelligence. A paper where we will investigate the other features is currently being prepared.

The exhibit's location in Germany may have been accompanied by cultural bias regarding stereotype content and exposure to ethnic minorities. The exhibit's depictions and variety of skin tones do not necessarily correspond to real human skin tones.

## 9 Conclusion

Our findings can be interpreted as supporting the action‐guiding effect of racial stereotypes. Adapting one's “perfect” child toward a social environment where stereotypes govern social interaction may benefit the child regarding fulfilling these stereotypes. However, it may prolong the same stereotypes and thus worsen the situation for discriminated groups [39]. Hence, we must be attentive to how individual technological interventions can damage valuable goods in systems composed of the complex relationship between social and technological factors [40]. Similar to genetic engineering, the appropriate way to enable children to live their “best life” is controversial. Before explicitly formulating any ‘moral obligations’ Museum of the Future Nuremberg [19], we should carefully evaluate the potential harm that may emerge when people employ a hyper‐individualistic perspective on this question.

## Ethics Statement

This study was reviewed and approved by the Ethics Committee of the University of Bamberg, Germany (Dossier number 2023‐07/31).

## Conflicts of Interest

The authors declare no conflicts of interest.

## Supporting information

Ethnicity Supplement FINAL.

## Data Availability

Given the agreement of our cooperation partner, data will be made available upon reasonable request. This decision was made due to ongoing research with the data set and data sharing policy of the museum. After our projects have finished, data is likely to be made public. Requests for validation of published findings or those under review will always be accepted for this purpose alone. Permission to analyze the presented data was kindly granted by the *Deutsches Museum Nürnberg – Das Zukunftsmuseum*, Nuremberg, Germany.

## Endnotes

1 A paper investigating these two hypotheses and discussing the general theoretical background in the context of normative gender stereotypes is Döbler and Carbon [28]. Limitations, thereotical background and analysis plan are highly alike.

2 For critical comments on Sarrazin's primary islamophobic rhetoric see Littau [41] and for a critique on the Bell curve see Heckman [42].

3 Although the exhibit did communicate most common choices of manipulated traits after completion, it did not conditioned them on skin color or gender. Hence, the exhibit did not reproduced stereotype content by communicating peer choices.

4 See Döbler and Carbon [28]. Here we also explore different motivations for creating a stereotype adhering child, for instance, high genetic essentialism.
