# Supplementary material for: Exploring the Hypothetical Impact of Genetic Engineering on Ethnicity: An Analysis of a Large‐Scale Data Set Retrieved From a Museal Setting
Source: Bioethics. 2025 Jun 25;40(1):94–103. doi: 10.1111/bioe.70005 (PMC12710675; doi:10.1111/bioe.70005)
Supplement: Supplementary file 1 — Ethnicity Supplement FINAL. [file BIOE-40-94-s001.docx]

# Supplement

## Predicting skin color change

| **Table S1. Skin color change coefficients** | | | | |
| --- | --- | --- | --- | --- |
| **Randomly assigned initial skin color** | **n** | **Magnitude of the skin color change** | | |
| **(hex color codes)** |  | **Log-Odds (Median)** | **CI (95%)** | **MPE** |
| Type 1 #F2D3AF | 3427 | 0.39 | 0.29 .. 0.50 | 100% |
| Type 2 #DEBB8D (Reference) | 3424 | 0.86 | 0.79 .. 0.93 | 100% |
| Type 3 #613928 | 3443 | 1.06 | 0.94 .. 1.18 | 100% |
| Type 4 #361103 | 3347 | 0.95 | 0.83 .. 1.07 | 100% |
| Observations | 13641 |  |  |  |
| Note. Color of the column and hex code represent randomly assigned initial skin color. CI = credible interval. MPE is a Maximal Probability of Effect: A probability that a parameter is strictly positive or negative. It is a measure of statistical significance of the effect with higher values indicating stronger evidence for statistically significant results. Type 2 was the reference category, so to obtain estimations for other types, linear combination of log-odds values must be done. | | | | |

## Predicting chosen color

family: zero_one_inflated_beta

Links: mu = logit; phi = log; zoi = logit; coi = logit

Formula: Choices.looks.skinColorSliderValue_rs ~ Nature.looks.skinColor

phi ~ Nature.looks.skinColor

zoi ~ Nature.looks.skinColor

coi ~ Nature.looks.skinColor

Data: skin_color_slider_01 %>% skin_color_slider_01 %>% filter (Changed.looks.skin == TRUE) (Number of observations: 10,954)

Draws: 4 chains, each with iter = 10,000; warmup = 5,000; thin = 1;

total post-warmup draws = 20,000

Regression Coefficients:

| **Parameter** | **Median** | **95% CI** | **pd** | **Rhat** | **ESS** |
| --- | --- | --- | --- | --- | --- |
| (Intercept) (Type 2) | -0.38 | [-0.42, -0.35] | 100% | 1 | 17405 |
| phi_Intercept (Type 2) | 1.81 | [1.75, 1.86] | 100% | 1 | 16815 |
| zoi_Intercept (Type 2) | -2.03 | [-2.16, -1.91] | 100% | 1 | 18023 |
| coi_Intercept (Type 2) | -0.26 | [-0.50, -0.02] | 98.52% | 1 | 19215 |
| Nature.looks.skinColorType 4 | -0.01 | [-0.05, 0.03] | 68.33% | 1 | 18386 |
| Nature.looks.skinColorType 3 | 0.02 | [-0.02, 0.06] | 81.91% | 1 | 18430 |
| Nature.looks.skinColorType 1 | -0.13 | [-0.17, -0.08] | 100% | 1 | 18880 |
| phi_Nature.looks.skinColorType 4 | 0.03 | [-0.04, 0.11] | 80.87% | 1 | 17875 |
| phi_Nature.looks.skinColorType 3 | 0.08 | [0.01, 0.15] | 98.21% | 1 | 18479 |
| phi_Nature.looks.skinColorType 1 | -0.06 | [-0.14, 0.02] | 93.45% | 1 | 18723 |
| zoi_Nature.looks.skinColorType 4 | -0.25 | [-0.43, -0.08] | 99.79% | 1 | 18735 |
| zoi_Nature.looks.skinColorType 3 | -0.13 | [-0.30, 0.04] | 92.67% | 1 | 19715 |
| zoi_Nature.looks.skinColorType 1 | -0.03 | [-0.20, 0.15] | 62.16% | 1 | 19736 |
| coi_Nature.looks.skinColorType 4 | -0.82 | [-1.20, -0.47] | 100% | 1 | 19761 |
| coi_Nature.looks.skinColorType 3 | -0.45 | [-0.79, -0.11] | 99.56% | 1 | 19771 |
| coi_Nature.looks.skinColorType 1 | 0.05 | [-0.27, 0.38] | 62.49% | 1 | 19209 |

## Zero-one inflated beta regression of designed intelligence

Family: zero_one_inflated_beta

Links: mu = logit; phi = log; zoi = logit; coi = logit

Formula: Choices.talents.intelligence ~ Choices.looks.skinColorSliderValue

phi ~ Choices.looks.skinColorSliderValue

zoi ~ Choices.looks.skinColorSliderValue

coi ~ Choices.looks.skinColorSliderValue

Data: skin_color_slider %>% filter(Changed.talents.intelligence == TRUE) (Number of observations: 10,737)

Draws: 4 chains, each with iter = 10,000; warmup = 5,000; thin = 1;

total post-warmup draws = 20,000

Regression Coefficients:

| Parameter | Median | 95% CI | pd | Rhat | ESS |
| --- | --- | --- | --- | --- | --- |
| (Intercept) | 1.12 | [1.09, 1.14] | 100% | 1.000 | 26374.00 |
| phi_Intercept | 1.66 | [1.63, 1.70] | 100% | 1.000 | 28459.00 |
| zoi_Intercept | -0.37 | [-0.41, -0.32] | 100% | 1.000 | 35160.00 |
| coi_Intercept | 1.93 | [1.84, 2.03] | 100% | 1.000 | 30813.00 |
| Choices.looks.skinColorSliderValue | -0.08 | [-0.13, -0.03] | 99.93% | 1.000 | 30892.00 |
| phi_Choices.looks.skinColorSliderValue | -0.16 | [-0.22, -0.10] | 100% | 1.000 | 30740.00 |
| zoi_Choices.looks.skinColorSliderValue | 0.29 | [0.21, 0.36] | 100% | 1.000 | 30271.00 |
| coi_Choices.looks.skinColorSliderValue | -0.53 | [-0.69, -0.38] | 100% | 1.000 | 30433.00 |

Pd = probability of direction / Maximal Probability of Effect

## Beta regression of designed intelligence

Family: beta

Links: mu = logit; phi = log

Formula:

Choices.talents.intelligence ~ Choices.looks.skinColorSliderValue

phi ~ Choices.looks.skinColorSliderValue

Data: skin_color_slider %>% filter(Changed.talents.intelligence == TRUE,

Choices.talents.intelligence > 0,

Choices.talents.intelligence < 1), (Number of observations: 6,488)

Draws: 4 chains, each with iter = 10,000; warmup = 5,000; thin = 1;

total post-warmup draws = 20,000

Regression Coefficients:

| **Parameter** | **Median** | **95% CI** | **pd** | **Rhat** | **ESS** |
| --- | --- | --- | --- | --- | --- |
| (Intercept) | 1.12 | [1.09, 1.14] | 100% | 1 | 17256 |
| phi_Intercept | 1.66 | [1.63, 1.70] | 100% | 1 | 17785 |
| Choices.looks.skinColorSliderValue | -0.08 | [-0.13, -0.03] | 99.92% | 1 | 18513 |
| phi_Choices.looks.skinColorSliderValue | -0.16 | [-0.22, -0.10] | 100% | 1 | 18551 |

Pd = probability of direction
